# Soils of two Antarctic Dry Valleys exhibit unique microbial community structures in response to similar environmental disturbances

**DOI:** 10.1186/s40793-024-00587-0

**Published:** 2024-07-26

**Authors:** Mafalda S. Baptista, Charles K. Lee, Maria R. Monteiro, Luís Torgo, S. Craig Cary, Catarina Magalhães

**Affiliations:** 1 Interdisciplinary Centre of Marine and Environmental Research, University of Porto, Matosinhos, Portugal; 2 International Centre for Terrestrial Antarctic Research, University of Waikato, Hamilton, New Zealand; 3 School of Science, University of Waikato, Hamilton, New Zealand; 4 Faculty of Sciences, University of Porto, Porto, Portugal; 5 Ocean Frontier Institute, Dalhousie University, Halifax, NS Canada

**Keywords:** Antarctica, McMurdo Dry Valleys, Microbial communities, Selective pressure

## Abstract

**Background:**

Isolating the effects of deterministic variables (e.g., physicochemical conditions) on soil microbial communities from those of neutral processes (e.g., dispersal) remains a major challenge in microbial ecology. In this study, we disturbed soil microbial communities of two McMurdo Dry Valleys of Antarctica exhibiting distinct microbial biogeographic patterns, both devoid of aboveground biota and different in macro- and micro-physicochemical conditions. We modified the availability of water, nitrogen, carbon, copper ions, and sodium chloride salts in a laboratory-based experiment and monitored the microbial communities for up to two months. Our aim was to mimic a likely scenario in the near future, in which similar selective pressures will be applied to both valleys. We hypothesized that, given their unique microbial communities, the two valleys would select for different microbial populations when subjected to the same disturbances.

**Results:**

The two soil microbial communities, subjected to the same disturbances, did not respond similarly as reflected in both *16S rRNA* genes and transcripts. Turnover of the two microbial communities showed a contrasting response to the same environmental disturbances and revealed different potentials for adaptation to change. These results suggest that the heterogeneity between these microbial communities, reflected in their strong biogeographic patterns, was maintained even when subjected to the same selective pressure and that the ‘rare biosphere’, at least in these samples, were deeply divergent and did not act as a reservoir for microbiota that enabled convergent responses to change in environmental conditions.

**Conclusions:**

Our findings strongly support the occurrence of endemic microbial communities that show a structural resilience to environmental disturbances, spanning a wide range of physicochemical conditions. In the highly arid and nutrient-limited environment of the Dry Valleys, these results provide direct evidence of microbial biogeographic patterns that can shape the communities’ response in the face of future environmental changes.

**Supplementary Information:**

The online version contains supplementary material available at 10.1186/s40793-024-00587-0.

## Background

Antarctic ice-free regions make up only ca. 1% of the land and yet they harbour almost its entire known terrestrial diversity [1]. Microbial communities are the terrestrial dominant forms of life, and the ones who drive key biological functions within these regions [2, 3]. The patchy distribution of ice-free regions physically isolates different areas, and the severity of the environmental conditions selects for the microbial populations which are more adapted to the local geochemistry [4].

The McMurdo Dry Valleys, in the Transantarctic Mountains, are one of those ice-free regions, composed of a mosaic of arid soils, ephemeral streams, lakes and glaciers [5]. The Dry Valleys have been described as one of the few environments where abiotic factors clearly drive the diversity and abundance of the microbial communities [6–8]. In several Dry Valleys a pronounced spatial heterogeneity of microbial communities has been linked to abiotic factors [9, 10]. Differences in soil geochemical properties like pH, ionic composition, carbon and nitrogen content, or water availability, are usually suggested as the major driving forces for the observed patterns of microbial distribution [11–15]. In these oligotrophic soils water content may be below 2%, organic carbon content below 0.5%, and levels of salinity can be generally high, imposing strong limitations on the survival, prevalence and distribution of soil microbiota [16, 17].

The harsh conditions of the Dry Valleys make its microbial communities an extreme case of environmental adaptability [18]. Because microbial communities’ response to environmental disturbance remains difficult to measure, studying these simplistic communities can greatly contribute to testing fundamental concepts. Factors determining species distribution patterns are relatively well established in macroecology. In contrast, for microecology such factors are not so well known [19]. Attempts at extending ecological theory to microorganisms have been made, particularly focussing on the effect of environmental selection on community assemblages, with historical processes and evolutionary trends receiving much less attention [20].

Besides environmental factors, dispersal is another process that can play an important role in community assembly. However, the factors that govern the relative influence of deterministic or stochastic processes are still not fully understood [21]. In the Dry Valleys, microbial communities have been shown to respond rapidly to changes in environmental conditions. In a direct soil modification experiment (transplanting of a mummified seal to an untouched neighbouring site, altering the underlying soil environment by stabilizing temperatures, elevating relative humidity and reducing ultraviolet exposure) soil microbial communities underwent rapid and lasting changes [22]. Notwithstanding, there is still a lack of detailed understanding of how microbial diversity has the necessary plasticity to respond to environmental changes. This becomes even more pressing in the face of global climate change, as the Dry Valleys microbial communities will be affected by inevitable upcoming changes. With climate change and the expected increase in connectivity across the continent the impacts on local biodiversity are unknown but could lead to an increase in biotic homogenization and loss in diversity [23].

Here we present the results from laboratory-controlled experiments to study the turnover in soils microbial communities when environmental disturbances are applied. Two McMurdo Dry Valleys, Beacon and Miers, were selected based on their contrasting weather patterns and edaphic characteristics. Miers Valley (MV), with an altitude of 171 m.a.s.l. (metres above sea level), is more subject to a maritime influence, whereas Beacon Valley (BV), at an altitude of 1376 m.a.s.l., is characterised by lower temperatures and stronger winds. In addition, MV soils have been described as sustaining higher carbon (C) and nitrogen (N) content [10] and also higher microbial biomass and diversity, compared to BV soils [7, 24]. We applied severe environmental disturbances to Beacon and Miers soils by increasing water availability, C and N content, and copper and sodium salts.

Microbial community structure was analysed up to two months, in each disturbance, by sequencing the V4 region of the *16S rRNA* gene, and the corresponding transcripts, to generate amplicon sequence variants (ASVs). This allowed uncovering the populations present in each sample and their potentially metabolically active fraction. Results were analysed to determine whether the biogeographic patterns seen at Beacon and Miers Valleys could also be seen as a result of the same disturbance applied. Given that the microbial communities of the two Dry Valleys were initially unique, we hypothesized that in the face of environmental changes the biogeographic patterns were likely to be maintained.

## Methods

### Dry Valleys soil samples

**Fig. 1:**
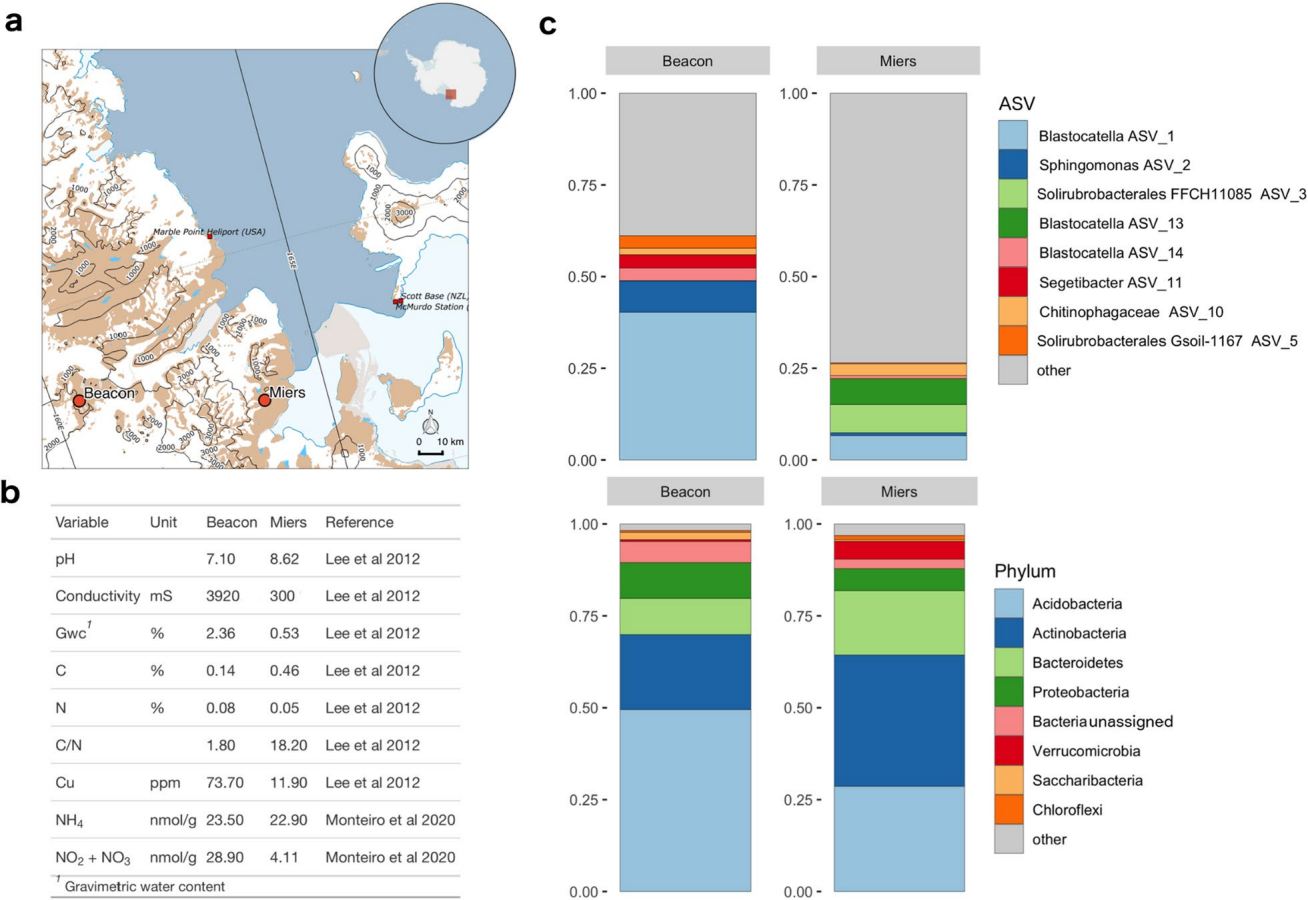
Characterisation of Beacon and Miers Valleys. **a** Location of Beacon and Miers Valleys within the Dry Valleys, in Victoria Land (shown as a rectangle on the inset map of Antarctica). **b** Soil geochemical data at Beacon and Miers Valleys, that informed and guided the disturbance experiment setup. **c** Community composition based on the *16S rRNA* genes, shown as proportions of total reads, with ASVs on the top panel annotated to the lowest assigned taxonomic rank, and on the bottom panel annotated to phyla. Taxonomy was assigned with SILVA v128. The eight most abundant taxonomic ranks are shown, and all other taxa are grouped under “other”

Soil samples from Beacon Valley (− 77.872017°, 160.495417°) and Miers Valley (− 78.091433°, 163.808983°) were collected at the McMurdo Dry Valleys, Victoria Land, Antarctica (Fig. 1a). Collection took place during a 2013 expedition under the New Zealand Terrestrial Antarctic Biocomplexity Survey (nzTABs, https://ictar.aq/nztabs-science/). The top 2 cm were aseptically collected with a sterile trowel. Samples were transported on ice to Scott Base (stored at − 30 °C) and then to the University of Waikato, New Zealand, where they were kept at − 80 °C until being used in 2015. Previous works informed and guided the setup of the disturbance experiment (Fig. 1b). BV soils displayed higher electrical conductivity, gravimetric water content and nitrite plus nitrate (NO_2_^−^ + NO_3_^−^) concentrations [10, 25]. Inversely, the C/N ratio was higher at MV [10]. In terms of elemental composition both soils were similar, except for Cu which showed a concentration at BV six times higher than at MV [10].

### Disturbance experiment set up

Undisturbed soils from each valley were used to set up the disturbance experiment, consisting of six treatments. Soils were thawed, sieved (2 mm) and homogenised, and an aliquot taken for DNA extraction (T0). Disturbances were set up with ca. 40 g of soil amended with 13 mL solutions in sterile 50 mL falcon tubes. To mimic an increase in Cu a 200 µM copper chloride (CuCl_2_) solution was employed; to mimic an increase in N (low and high) solutions of 4 or 15 µM ammonium chloride (NH_4_Cl) were employed, respectively; to mimic an increase in C and organic matter a 300 µM glucose (C_6_H_12_O_6_) solution was employed; to mimic an increase of 3000 µS/cm in electric conductivity a solution with the appropriate concentration of sodium chloride (NaCl) was employed; and to mimic an increase in water availability Milli-Q water was employed. All the solutions were prepared with autoclaved Milli-Q water, using reagents *pa* grade or equivalent, in lab ware autoclaved and decontaminated by soaking in a 20% HCl (37%) bath overnight. The soils were aerobically exposed at 4 °C, in the dark. After T1 (1 day), T2 (28 days) and T3 (65 days for BV or 68 days for MV), one falcon tube per treatment was destructively sampled for DNA and RNA extraction.

### Nucleic acid extraction, library construction and sequencing

Total RNA was extracted from 15 g of soil immediately after sampling (PowerSoil RNA isolation kit, MoBio), treated with 1U l^−1^ of DNase (TURBO DNase, Ambion Life Technologies) followed by PCR (see below) to check for DNA contamination. Reverse transcription was then performed using the Thermoscript RT-PCR kit with random primers (ThermoFisher Scientific). DNA was extracted from 0.8 to 1 g of soil using a modification of the CTAB extraction protocol [24]. DNA and cDNA were quantified using Qubit 2.0 HS assay (ThermoFisher Scientific) and then used to amplify the V4 region of the *16S rRNA* gene, using the primers 515F (5′-GTGYCAGCMGCCGCGGTAA) [26] and 806R (5′-GGACTACNVGGGTWTCTAAT) [27], acquired from the Earth Microbiome Project as fusion primers with a unique barcode and an Ion Torrent specific adapter sequence. Triplicate 25 µl reactions were set up with 1 ng of nucleic acid, 10 µM of each primer, 0.2 mM dNTPs, 1U of Platinum Taq (Invitrogen), 10 × Platinum Taq PCR buffer, 3 mM MgCl2 and 0.4 mg/ml of BSA. The master mix was treated with ethidium monoazide (EMA) to minimise amplification of extracellular DNA (1 µl of EMA to 100 µl of master mix). The amplification was performed with an initial denaturation step of 3 min at 94 °C, followed by 30 cycles of denaturation at 94 °C for 45 s, annealing at 50 °C for 60 s and extension at 72 °C for 90 s. A final extension was carried out for 10 min at 72 °C. The PCR products were pooled, checked in a 1% agarose gel, and cleaned with the SPRIselect kit (Beckman Coulter).

An equimolar library was constructed, after DNA quantification (Qubit), and emulsion PCR was carried out using the Ion OneTouch 2 System (Life Technologies), and the Ion PGM Template OT2 400 Kit (Life Technologies), to produce positive enriched Ion Sphere Particles (ISPs) with 400 base-pair average clonal fragments. Sequencing of the enriched, template-positive ISPs was carried out on an Ion 318 Chip v2 (Life Technologies) using the Ion Torrent PGM system (Life Technologies) and the Ion PGM Sequencing 400 Kit (Life Technologies) at the Waikato DNA Sequencing Facility (University of Waikato, New Zealand).

### Sequence processing

Sequence processing and analysis was performed in R v.4.1.0 (www.R-project.org). Demultiplexed Ion Torrent fastaq files, sequenced in three different runs, were imported using package DADA2 v.1.16 [28]. Filtering, trimming, error rates learning and ASV inference were performed with default settings, using the additional parameters recommended for Ion Torrent (https://benjjneb.github.io/dada2/faq.html#can-i-use-dada2-with-my-454-or-ion-torrent-data). ASV tables obtained for each run were then merged. Chimeras were removed with the *removeBimeraDenovo* function using the method “consensus”. Taxonomy was assigned with the function *assignTaxonomy* and the function *addSpecies* to assign genus-species binomials, using the DADA2 implementation of the naive bayesian classifier and DADA2-formatted reference databases (https://benjjneb.github.io/dada2/training.html). The reference databases were SILVA v128 and GTDB r86. A phylogenetic tree was built upon sequence alignment with package DECIPHER v.2.20.0 [29], using the function *AlignSeqs*. Distance was computed with function *dist.ml* and an unrooted tree was built with the neighbour joining method (function *treeNJ*), maximising the likelihood for a gamma distribution (function *pml*), using the GTR model, in package phangorn v.2.8.0 [30].

The sequenced data set comprised 63 samples, 38 DNA- and 25 RNA-based, respectively. This was due to not enough RNA being extracted from most BV samples. Two RNA-based samples were removed, after inspecting the reads quality profiles and determining the mean quality score at each position to be too low (water disturbance at T3, for both BV and MV). This resulted in a data set with a median number of sequences of 19,220 (ranging from 4833 to 48,626), and a total of 2925 ASVs. No obvious skewing could be seen in terms of number of sequences between both valleys or between DNA or RNA-based samples (Supplementary Fig. S1, Table S1). The sequence data, the taxonomy and the phylogenetic tree were combined in a single object with phlyoseq (v.1.36.0) [31]. Sequences assigned by SILVA v128 to eukaryotes, mitochondria and chloroplast were removed; this retained 96% of the ASVs. Additionally, the two RNA-based samples for the undisturbed soil were also removed, as they had been kept without a RNA stabiliser, prior to the laboratory-controlled experiment. A principal coordinates analysis (PCoA) was employed (as below) to compare the full data set with a data set filtered for ASVs that do not appear more than 2 times in at least 2 samples (retaining 45% of ASVs). The percentage of variance explained by the first two axes was compared in both data sets and the difference was considered negligible (Supplementary Fig. S2). Removing low abundance ASVs may be inappropriate to fully characterise abundance and diversity, if the community composition is severely changed, which was not the case.

### Analysis and visualisation

Plots were created in R v.4.1.0 with packages tidyverse v.1.2.1, microViz v.0.7.10 and gt v.0.2.2, and edited in Gimp v.2.8.14 (www.gimp.org/). A map of the sampling sites was created with QGIS v2.8.2 (https://www.qgis.org/) and Quantarctica v3 [32].

The initial communities at Beacon and Miers Valley were compared with a bar plot showing the most abundant taxonomic ranks. Differences in community composition between both valleys were assessed with a PCoA, for which UniFrac distances were calculated with phyloseq, on a centred log-ratio transformed ASV data set (function *clr* in microbiome v.1.14.0). Hereafter, the PCoA first axis was plotted against the number of days after disturbance. A permutational analysis of variance (PERMANOVA) was used to check for differences in communities between valleys, time and disturbance, after testing that samples did not differ significantly in their dispersion, by an analysis of multivariate homogeneity (PERMDISP). Both were performed in vegan (v.2.5.7) [33] with functions *adonis2* and *betadisper*, respectively, using 999 permutations and adjusting for sample bias.

To evaluate differences in DNA- and RNA-based community composition, the centred log-ratio transformed UniFrac distance of each DNA-RNA sample pair was computed within the PCoA ordination space, following the equation in [34]. The maximum number of PCoA dimensions was chosen by employing the axes that cumulatively explained 70% of the variation (n = 9), and that upon inspection of a scree plot were shown to capture the essential part of the variation. For each DNA-RNA pair, differences were retrieved for the employed dimensions, powered to two, and summed. The sum was square rooted to determine the overall distance for each disturbance.

The response to different disturbances was assessed by a hierarchical cluster analysis, performed on the centred log-ratio transformed ASV data set, using Euclidean distances and the Ward method (function *ward.D2* in stats v.4.1.0). For each valley and nucleic acid type the ten most abundant ASVs were then visualised with a heatmap. This cut off was chosen to allow for easy visualisation. To assess the response over time, the cumulative sum scaling (CSS) was used to transform the ASV data set, using default values (metagenomeSeq v.1.36.0) [35]. The communities were then visualised with ternary plots (ggtern v.3.3.5) [36], for each valley and nucleic acid type, with a two-dimensional kernel density estimation displayed as contours.

The response of the rare biosphere was assessed by looking at the ASVs with a relative abundance < 0.1%, a commonly used cut-off [37]. The ability to recruit bacterial populations from the seed bank was inspected over time, for each disturbance, by selecting the ASVs that cumulatively showed less than 0.1% relative abundance at T0 (deemed rare) and at least 0.1% relative abundance at subsequent time points (deemed not rare).

## Results

### Beacon and Miers Valleys characterization

Mirroring the edaphic differences between soils of both valleys, the microbial communities were shown to be distinct in abundance and composition (Fig. 1). For both valleys, four phyla accounted for most of the diversity, with Acidobacteria, Actinobacteria, Bacteroidetes and Proteobacteria representing ca. 90% of all ASVs. Highlighting the isolation of these communities, unassigned Bacteria also exhibited a high abundance (Fig. 1c, bottom panel).

Noticeably, ASVs assigned to the same taxonomic rank showed quite disparate abundances between valleys (Fig. 1c). Genus *Blastocatella* (Acidobacteria, Blastocatellaceae), the most abundant in both valleys, was predominantly assigned to ASV_1 and ASV_14 at Beacon Valley and to ASV_13 at Miers Valley. Interestingly, the GTDB taxonomy assigned these ASVs to a different Acidobacteria family, Pyrinomonadaceae. The fact that the most abundant ASV is taxonomically ambiguous up to the family rank is a good indicator of the lack of representativeness of these communities in databases and a measure of their uniqueness. Archaea showed a relative abundance of ca. 0.3%. They were assigned to the soil crenarchaeotic group in SILVA v128 and to *Nitrosocosmicus oleophilus* (GB_GCA_000802205.2) in GTDB r86.

Miers Valley showed roughly three times more ASVs than Beacon Valley (Supplementary Fig. S3). At Beacon Valley the eight most abundant ASVs accounted for more than 50% of the diversity, whereas at Miers Valley the eight most abundant ASVs accounted only for ca. 25% of the diversity (Fig. 1c). Overall, the Beacon Valley community was composed of a lower number of ASVs that tended to be overrepresented, whereas the Miers Valley community was composed of a more diverse set of not overabundant ASVs.

### Microbial community response on Beacon and Miers Valleys disturbed soils

**Fig. 2:**
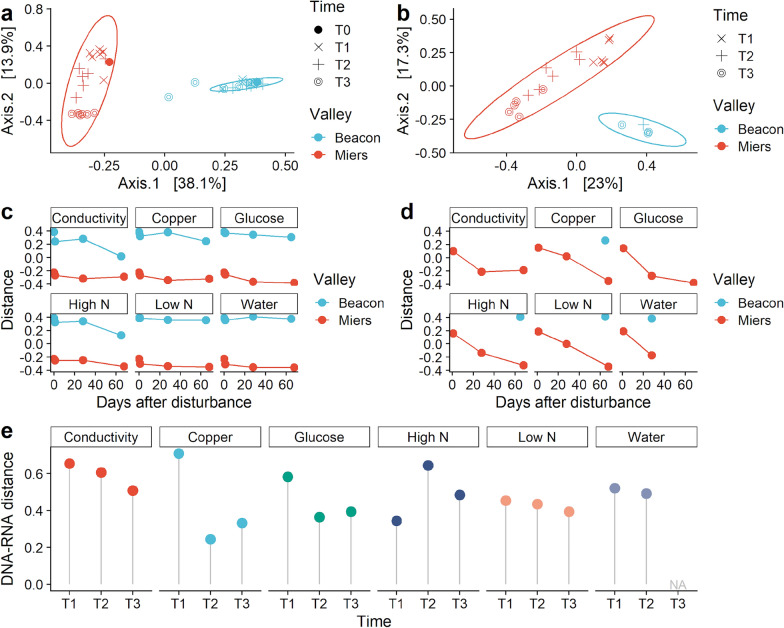
Comparison of the microbial community structures in disturbed soils from Beacon and Miers Valleys. PCoA of **a** *16S rRNA* genes and **b** *16S rRNA* transcripts performed on the UniFrac distances of a centred log ratio transformed ASVs data set. Ellipses are overlaid at the 0.95 level. **c, d** The UniFrac distances plotted over time, for the genes and transcripts data sets, respectively. **e** Pairwise distance between DNA- and RNA-based communities in Miers Valley, for each disturbance and time points. DNA-RNA distance was calculated from the PCoA, along the number of axes (n = 9) that cumulatively captured 70% of variance

After exposure for up to two months to different disturbances the microbial communities were still well separated according to their provenance from each valley. A PCoA showed this could be seen for both the *16S rRNA* genes (DNA) and transcripts (RNA) data sets (Fig. 2a, b). However, even if a PERMANOVA did show differences between both valleys, the PERMDISP showed a great dispersion of the results (Table 1).

**Table 1:** PERMANOVA and PERMDISP results

Nucleic acid	Data	PERMANOVA				PERMDISP		
		Df	F-statistic	R2	*P* value	Df	F-statistic	*P* value
DNA	Valley	1	20.421	0.362	0.001	1	27.169	0.001
	Disturbance	6	0.581	0.101	0.986	–	–	–
	Time	3	1.570	0.121	0.053	–	–	–
RNA	Valley	1	4.644	0.196	0.001	1	16.665	0.001
	Disturbance	5	0.770	0.204	0.911	–	–	–
	Time	2	2.685	0.230	0.001	2	3.142	0.075

When the results of the PERMANOVA were not indicative of difference the PERMDISP was not performed (–). For the RNA data set a correction for an unequal number of samples was applied

Still, the PCoA ordination suggested a temporal trend, with disturbances at T1 (1 day), T2 (28 days) or T3 (65 or 68 days for Beacon and Miers, respectively) clustering closer. To further inspect this trend the first axis of the PCoA was plotted versus the number of days after disturbance (Fig. 2c, d). For the *16S rRNA* genes data set, increases in distance were especially seen between the microbial communities of the undisturbed soil and after 1 day of disturbance (Fig. 2c). Afterwards, the distance between microbial communities changed very slightly over time. Conversely, for the *16S rRNA* transcripts data set, in Miers Valley, it was clear that the microbial community composition changed throughout time. Steep increases in distance could be seen for both high and low N disturbances and glucose (Fig. 2d), and more gradual increases could be seen for copper and conductivity disturbances. Lack of *16S rRNA* transcripts data for Beacon Valley precludes a similar analysis, but the available data are shown to emphasise that the samples for which RNA extraction was successful were mostly those disturbed for ca. 60 days, suggesting that an increase of microbial biomass took place throughout the laboratory-controlled experiment.

To determine how community assemblies differed when characterised by *16S rRNA* genes or transcripts, we inspected the distance between each DNA-RNA sample pair for each disturbance, over time (Fig. 2e). This was done for Miers Valley, where DNA and RNA-based samples were available. It showed that the largest distances were seen at T1, except for the high N disturbance where the largest distance was seen at T2. In terms of temporal trends, the distances became increasingly smaller as time went by, however, the copper and glucose disturbances showed larger distances at T3 than at T2. These results suggested that the different selective pressures drove changes in the bacterial populations at different paces.

### Microbial community response to the different environmental disturbances

**Fig. 3:**
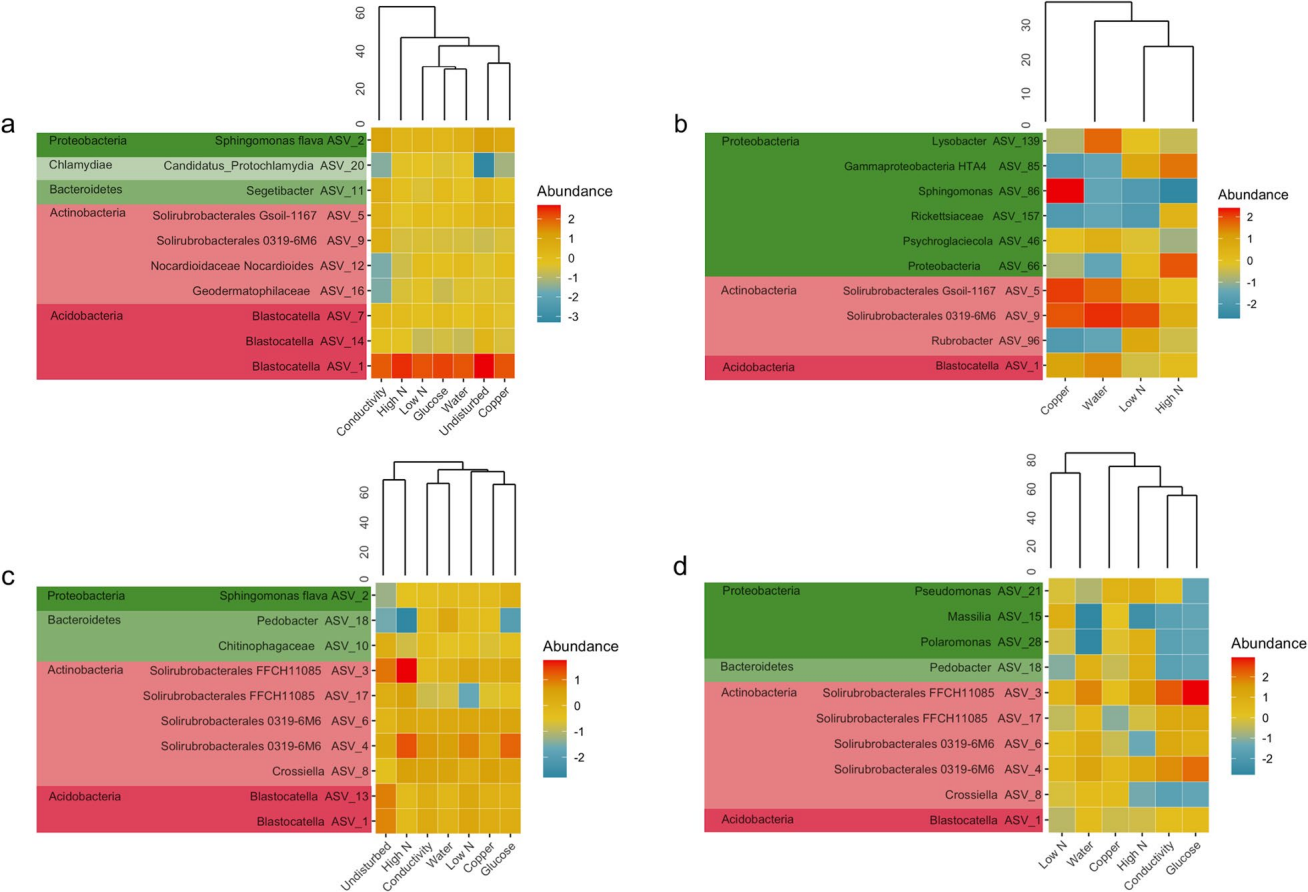
Clustering of the microbial communities for each disturbance, and heatmap of the ten most abundant ASVs in each of them. **a, b** Beacon Valley *16S rRNA* genes and *16S rRNA* transcripts data sets, respectively. **c, d** Miers Valley *16S rRNA* genes and *16S rRNA* transcripts data sets, respectively. For each valley and data set, disturbances were merged for all time points and ASV abundance was centred log-ratio transformed, before clustering was performed. The ten most abundant ASVs are shown with the lowest taxonomic rank assigned (SILVA v128) and grouped by phyla

A hierarchical cluster analysis was performed to inspect how the different disturbances were translated into differences in community composition. It showed that different bacterial populations responded to the same disturbances at Beacon and Miers valleys (Fig. 3). For the *16S rRNA* genes data set at Beacon Valley, the conductivity disturbance presented distinct bacterial populations abundances. Particularly noticeable was the similarity between undisturbed and disturbed soils (Fig. 3a). Contrastingly, at Miers Valley the undisturbed soil community clustered apart from the disturbed ones, the exception being the high N supplementation (Fig. 3c).

At Miers Valley, the *16S rRNA* transcripts data set did not follow the same clustering as the *16S rRNA* genes and showed low N and water disturbances having more similar bacterial populations than the remaining disturbances (Fig. 3c, d). At Beacon Valley, in spite of the low number of samples for the *16S rRNA* transcripts data set, a contrasting response between genes and transcripts data set could still be seen (Fig. 3a, b). However, due to the imbalance in RNA samples, no straightforward comparison could be drawn.

Heatmaps were employed to visualise the top ten bacterial populations in each disturbance. Noticeably, the most abundant populations were only partially overlapping between DNA- and RNA-based samples. For ASVs that were present in both data sets, changes in abundance were more noticeable for the transcripts than for the genes data set, suggesting that using RNA to capture the potentially metabolically-active fraction of the community was effective. For instance, at Miers Valley, genus *Crossiella* showed no changes in abundance for the genes data set, whereas for the transcripts data set a change in abundance was seen for the high N, conductivity and glucose disturbances (Fig. 3c, d). The use of RNA has also enabled capturing ASVs that were not highly abundant in the genes data set but were top abundant in the transcripts data, such as Proteobacteria ASVs assigned to *Pseudomonas*, *Massilia* and *Polaromonas* (Fig. 3c, d). For Beacon Valley this was also noticeable for a number of ASVs assigned to Proteobacteria (Fig. 3b). In fact, the combined data for both valleys highlights that Proteobacteria populations frequently responded to the disturbances in the transcripts data set, even if in the *16S rRNA* genes no response could be seen.

**Fig. 4:**
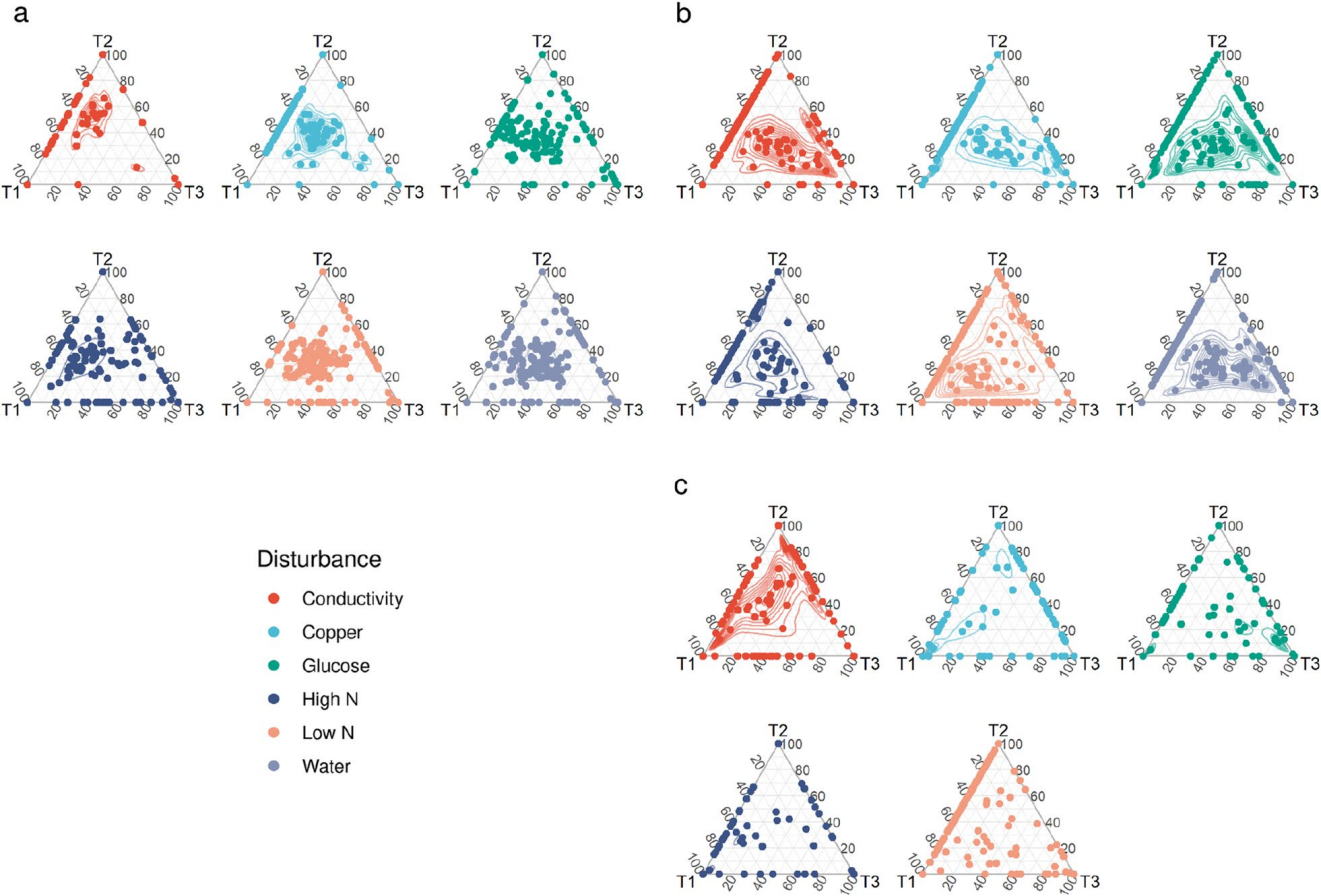
Temporal variation of bacterial populations in each disturbance. **a** Beacon Valley *16S rRNA* genes **b**, **c** Miers Valley *16S rRNA* genes and transcripts data sets, respectively. Time points correspond to 1 day (T1), 28 days (T2) and 65 or 68 days for Beacon and Miers, respectively (T3). The ASVs data set was transformed with the cumulative sum scaling to account for variation in total counts between samples. Each dot corresponds to an ASV; the density estimation was performed by kernel smoothing and is displayed as contours

The abundance of bacterial populations was followed throughout time, to determine temporal trends in response to different disturbances. For Beacon Valley the results showed the better part of the ASVs presented similar abundances throughout the ca. two-month experiment (Fig. 4a). Conductivity and copper disturbances showed a great number of ASVs that were only present at T1, but for the other disturbances bacterial populations present throughout time were more represented than bacterial populations specific to one time point (Fig. 4a).

In contrast, for Miers Valley a temporal trend could be seen (Fig. 4b, c). For all the disturbances a high number of ASVs were only present at T1. For glucose and water disturbances a great number of ASVs had high abundances at both T1 and T2, and for N disturbances a great number of ASVs also showed high abundances at T3 (Fig. 4b). For the *16S rRNA* transcripts no similarity with the genes data set could be seen (Fig. 4c). Conductivity showed ASVs with high abundances at both T1 and T2, however, for the remaining disturbances bacterial populations specific to one time point were more represented than bacterial populations present throughout time (Fig. 4c). This suggested that different disturbances activated specific bacterial populations, over time.

Overall, these results show a distinct behaviour between Beacon and Miers Valleys microbial communities, where the same selective pressures induced different temporal trends in bacterial populations. These results reinforce the idea that the initial community composition was prevalent in determining the outcome of the microbial assemblies in response to disturbances.

### Rare biosphere response to disturbance

**Fig. 5:**
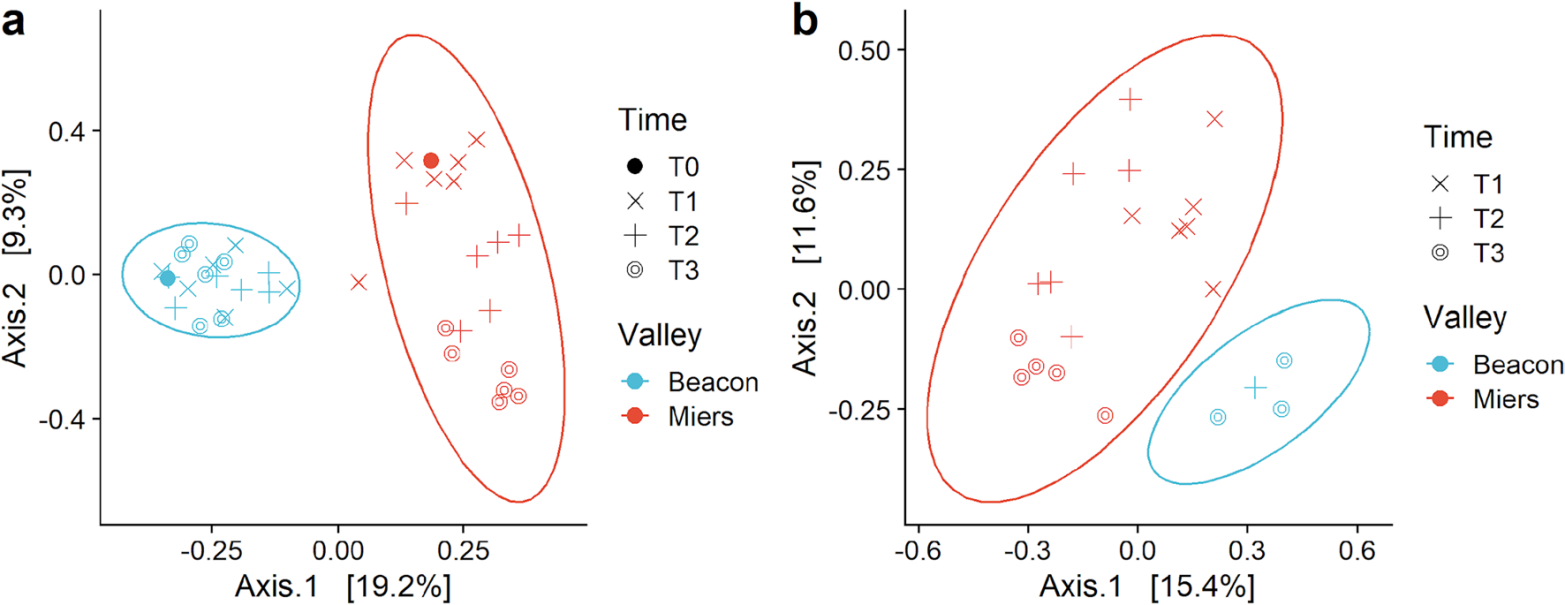
Comparison of the rare biosphere microbial communities in disturbed soils from Beacon and Miers Valleys. PCoA of **a** *16S rRNA* genes and **b** *16S rRNA* transcripts performed on the UniFrac distances of a centred log-ratio transformed ASVs data set. Ellipses are overlaid at the 0.95 level

Since the microbial seed bank can greatly influence microbial community turnover, we assessed its role by inspecting the rare biosphere of the microbial communities. We considered as rare the ASVs that showed a relative abundance < 0.1%. The rare biosphere responded in the same way as the whole community, and after exposure for up to two months to different disturbances the microbial communities from both valleys were still well separated (Fig. 5). As before, a PCoA showed this could be seen for both the *16S rRNA* genes (DNA) and transcripts (RNA) data sets (Fig. 5a, b).

To inspect how individual bacterial populations responded to the disturbances we considered rare ASVs that showed less than 0.1% relative abundance at T0 (undisturbed soil) and at least 0.1% relative abundance at disturbed soils (hence, not rare). This approach aimed at selecting the rare bacterial populations that responded to changes brought about by the environmental disturbances, over time.

**Fig. 6:**
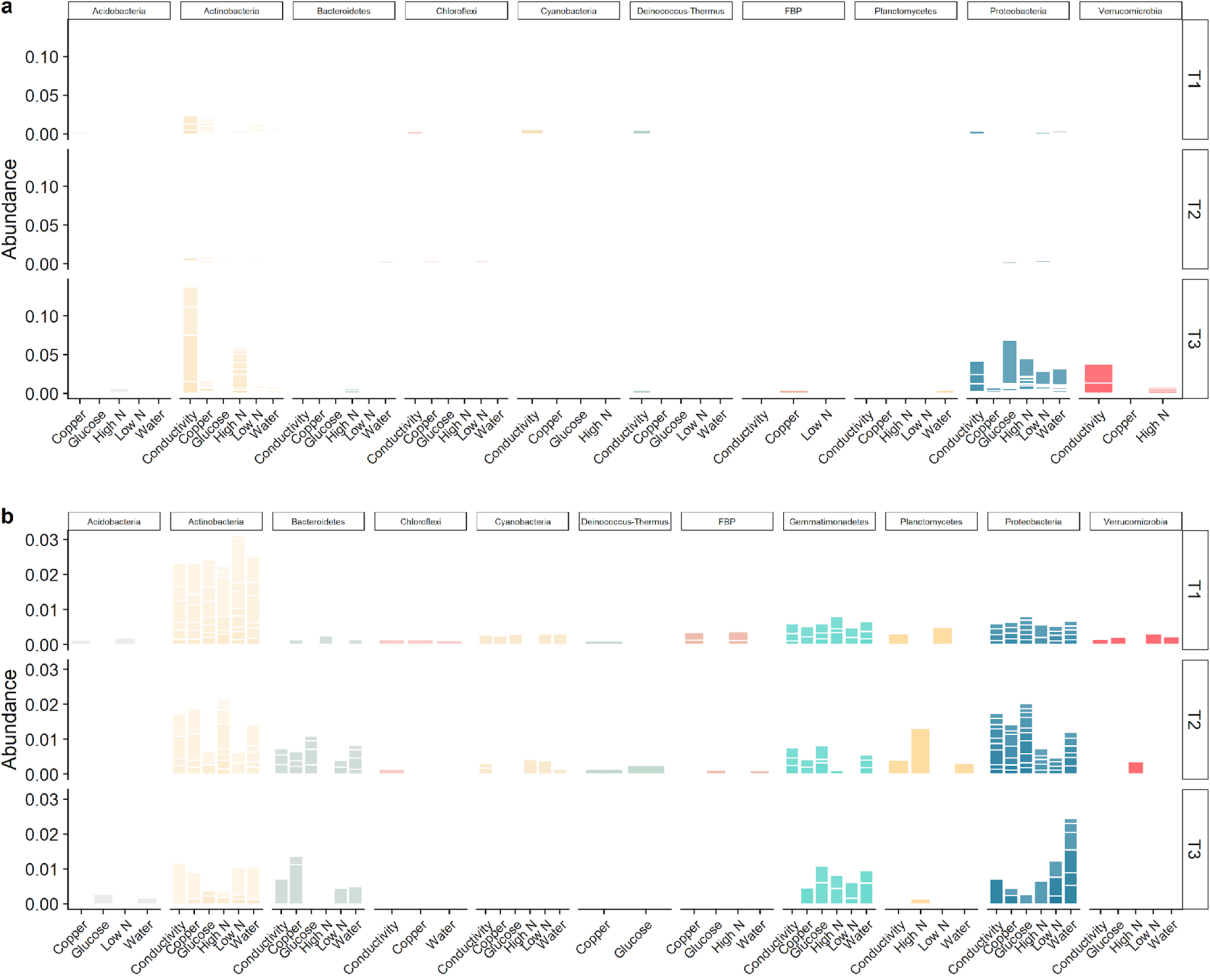
Rare biosphere response in **a** Beacon Valley and **b** Miers Valley. The *16S rRNA* genes data set was transformed to relative abundance and ASVs < 0.1% at T0 and > 0.1% at the remaining time points were retained. ASVs are shown grouped by phyla, each separated by a white line

The rare biosphere responded in a strikingly different way at Beacon and Miers Valleys (Fig. 6). At Beacon Valley several bacterial populations, especially assigned to Actinobacteria and Proteobacteria, showed a greater recruitment at T3 (Fig. 6a), whereas at Miers Valley most bacterial populations showed a greater recruitment at T1 and T2 (Fig. 6b). This suggested that Miers Valley populations responded rapidly and fleetingly when the disturbances were applied, while Beacon populations responded in a more lingering manner, or did not respond at all.

Inspecting individual bacterial populations another striking difference could be seen between both valleys. At Beacon most ASVs responded uniquely to specific disturbances, whereas at Miers most ASVs responded to every applied disturbance (Supplementary Fig. S4). The rare biosphere populations were overwhelmingly assigned to Actinobacteria, Bacteroidetes and Proteobacteria, in good agreement with the phyla distribution seen for the undisturbed soil. In sharp contrast was the absence of Acidobacteria and Saccharibacteria, which were well represented in the undisturbed soil, but practically missing from the rare biosphere recruited populations (Fig. 1c), and the response of bacterial populations assigned to Gemmatimonadetes, which were present at the undisturbed soil but were exclusively at Miers as rare biosphere (Fig. 6b).

## Discussion

The microbial communities of both valleys showed highly different taxonomic composition, reflected at higher and lower taxonomic ranks. A previous study, using automated ribosomal intergenic spacer analysis (ARISA), described highly localised bacterial communities in Beacon and Miers Valleys [10], which is in agreement with what was observed in this study using the V4 region of the *16S rRNA* gene (Fig. 1). In spite of the Dry Valleys being extremely harsh environments, which makes them exceptionally selective ecosystems, data from this study corroborate that microbial diversity varies greatly, and provides evidence that microbial communities exhibit biogeographic patterns within these arid environments.

The biogeography of soil microbial communities is thought to be primarily controlled by edaphic factors, hence soils with similar characteristics harbour similar microbial communities [38]. Furthermore, microbial spatial distributions are also determined by historical processes driven by dispersal, and disentangling the relative importance of these mechanisms remains difficult [39]. Both Beacon and Miers Valleys soils exhibited marked geochemical gradients, allowing the assumption that deterministic selection by environmental factors was driving the microbial heterogeneity. In this work, a laboratory-controlled experiment in which soils of both valleys were subjected to the same selective pressures, deterministic selection was dominating the microbial community assembly. However, these are historically isolated areas, where dispersal limitations could also influence the distribution of microorganisms [40], but still, other works have shown that environmental filtering can deterministically govern subsurface microbial community composition [41].

### The same selective pressures maintained distinct microbial communities at Beacon and Miers Valleys

The bacterial communities of both Beacon and Miers valleys did not converge, over a two-month period (Fig. 2). Still, legacy effects (when changes in community structure lag behind shifts in the environment) can shape the microbial community composition, and this can be a particularly important mechanism in areas where spatial isolation occurs [42]. Dormancy and taphonomy are confounding factors of microbial community assembly processes, making the interpretation of DNA-base patterns difficult [37]. In this work we have employed an ethidium monoazide PCR approach, in order to reduce the masking effects that relic DNA can have in the evaluation of community composition [43].

Beacon Valley, a harsher and more inhospitable environment, showed less diversity than Miers Valley (Fig. 1, Supplementary Fig. S3). It has been predicted that communities that are more diverse are more likely to contain species that can cope with a disturbance [44]. Under this assumption, Miers Valley microbial communities would have a higher ecological resilience (Figs. 2, 3). Furthermore, we have inspected the role of the seed bank in the assemblage of the communities, since recruitment from the rare biosphere can provide a reservoir of ecological resilience [37]. Miers Valley rare bacterial populations could be seen responding evenly to almost every disturbance applied, whereas at Beacon Valley populations deemed rare mostly responded to one disturbance (Fig. 6, Supplementary Fig. S4). These results suggest that this reservoir of ecological resilience could be seen at Miers Valley, but not at Beacon Valley.

### The biogeographic patterns seen at Beacon and Miers Valleys are likely to stand in face of upcoming environmental changes

In the Dry Valleys, microbial community structures have been shown to respond to changes in water and nutrient availability [22, 25, 45, 46]. Interestingly, in this work, and for Beacon Valley, no clear difference between undisturbed and disturbed soils could be seen (Fig. 3a). In addition, the most abundant ASVs presented similar relative abundances throughout the ca. two-month experiment (Fig. 4), except for the disturbance with increased conductivity. In fact, the greatest difference in community composition was seen in soils subjected to an increase in conductivity (Fig. 3a), possibly as a result of the high soil conductivity at Beacon Valley (Fig. 1b), which presupposes a community already adapted to high conductivity values.

For Miers Valley, however, the undisturbed soil community clustered apart from the disturbed ones, except for the high N disturbance (Fig. 3c). A clear temporal trend could be seen, both for the most abundant bacterial populations (Fig. 4) as for the rare biosphere (Fig. 6), and for the later a clear cut off for recruitment after ca. 28 days of disturbance could be seen. This suggested that exhausted the environmental conditions created by the disturbances, the rare biosphere would no longer play a role in responding to these selective pressures. In several ecosystems the rare biosphere is composed of conditionally rare taxa, i.e. taxa that occasionally, and usually in response to an environmental change, become very abundant [47, 48]. However, at Miers Valley the rare taxa never became over abundant, again highlighting the exceptional niche adaptation of the bacterial populations that thrive in the Dry Valleys extremely harsh ecosystems. In this study, mimicking a likely scenario in the near future, in which similar selective pressures may be applied to both valleys, the results show the unique microbial communities are likely to maintain their distinctiveness.

### Community characterization by RNA elucidated the response to different selective pressures

To further inspect how distinct selective pressures determined the assembly and successional dynamics of these microbial communities we employed an RNA-based approach. Although the number of ribosomal transcripts can vary, RNA-based approaches can still provide more certainty regarding the viability and activity of the microbial cells than DNA-based approaches [49]. This was successful for Miers Valley, and allowed comparing the response of DNA and RNA-based samples to different disturbances. Looking at the response of specific bacterial populations, the highest diversity of potentially metabolically active ASVs was seen for phyla Actinobacteria and Proteobacteria, with other highly DNA abundant phylum in the undisturbed soil, such as Acidobacteria, conspicuously absent (Fig. 3). Other works looking at the increase of water and organic matter availability in Antarctic soils have shown similar phyla distributions [22, 46] particularly when analysing the potentially metabolically active fraction [45].

Differences between DNA and RNA-based communities showed a temporal trend (Fig. 2) which was interpreted in terms of potential metabolic pathways that may be involved in responding to these disturbances. Copper and sodium salts can be readily uptake by specific and nonspecific channels taking advantage of their ubiquity in bacterial cells [50, 51]. Glucose uptake depends on a regulatory network that has a modular organisation, in which several transcriptional factors integrate different signals to respond to the presence of glucose [52]. Microbes in soil are typically limited by C, so when glucose is added it stimulates growth and activity, by increasing the transcription of genes associated with the N regulatory network, specifically the NH_4_^+^ conversion into amino acids [53]. However, at Miers Valley the extremely low content of ammonium in the soil (Fig. 1) could have dictated that the increase in glucose did not result in a rapid ammonium conversion, and therefore the 28 days delay in responding to high N disturbances. In addition, N supplied as NH_4_Cl is uptake by very specific bacterial populations involved in biochemical pathways of the nitrogen-cycling network [54], which could further account for the lag in time for the overall community to respond. As for the low N addition, the similarity of the results with the water addition suggests the response of the communities should be primarily interpreted as a response to water addition, known to be a key factor in determining community structure [46]. Therefore, under disturbed conditions the community structure provides valuable insight for predicting ecosystem processes.

## Conclusions

This study provided direct evidence that bacterial communities, inhabiting geochemically disparate soils of two Dry Valleys, present distinct patterns of diversity and will most likely present dissimilar structures in response to sudden and harsh environmental disturbances. Both *16S rRNA* genes and transcripts analysis supported this conclusion, and this could be seen for abundant bacterial populations as well as for the rare biosphere. These findings show that environmental selection within the Dry Valleys might not overcome the historical isolation of those microbial communities, making them vulnerable to invasion by generalist species under upcoming climate change. Although our experimental results hint at metabolically divergent assemblies between Beacon and Miers Valley, further insights into the function of these communities will prove invaluable to enhance our understanding of the functioning of these cold deserts of Antarctica.

### Supplementary Information

Supplementary material 1.

## Data Availability

Sequences are deposited under the SRA accession number PRJNA527658. The code for reproducing the sequence and data analysis is available at https://github.com/msbaptista/MDV-16S-disturbance
