# Supplementary material for: Soils of two Antarctic Dry Valleys exhibit unique microbial community structures in response to similar environmental disturbances
Source: Environ Microbiome. 2024 Jul 26;19:52. doi: 10.1186/s40793-024-00587-0 (PMC11282855; doi:10.1186/s40793-024-00587-0)
Supplement: Supplementary file 1 — Supplementary material 1. [file 40793_2024_587_MOESM1_ESM.docx]

**Soils of two Antarctica Dry Valleys exhibit unique microbial communities structures in response to similar environmental disturbances**

Mafalda S. Baptista^1,2^, Charles K. Lee^2,3^, Maria Monteiro^3^, Luís Torgo^4,5^, S. Craig Cary^2,3^ * Catarina Magalhães^1,4,5^ *

^1^ Interdisciplinary Centre of Marine and Environmental Research, University of Porto, Matosinhos, Portugal

^2^ International Centre for Terrestrial Antarctic Research, University of Waikato, Hamilton, New Zealand

^3^ School of Science, University of Waikato, Hamilton, New Zealand

^4^ Faculty of Sciences, University of Porto, Porto, Portugal

^5^ Ocean Frontier Institute, Dalhousie University, Halifax, Nova Scotia, Canada

Corresponding authors:

* caryc@waikato.ac.nz

* cmagalhaes@ciimar.up.pt

**Supplementary figures**


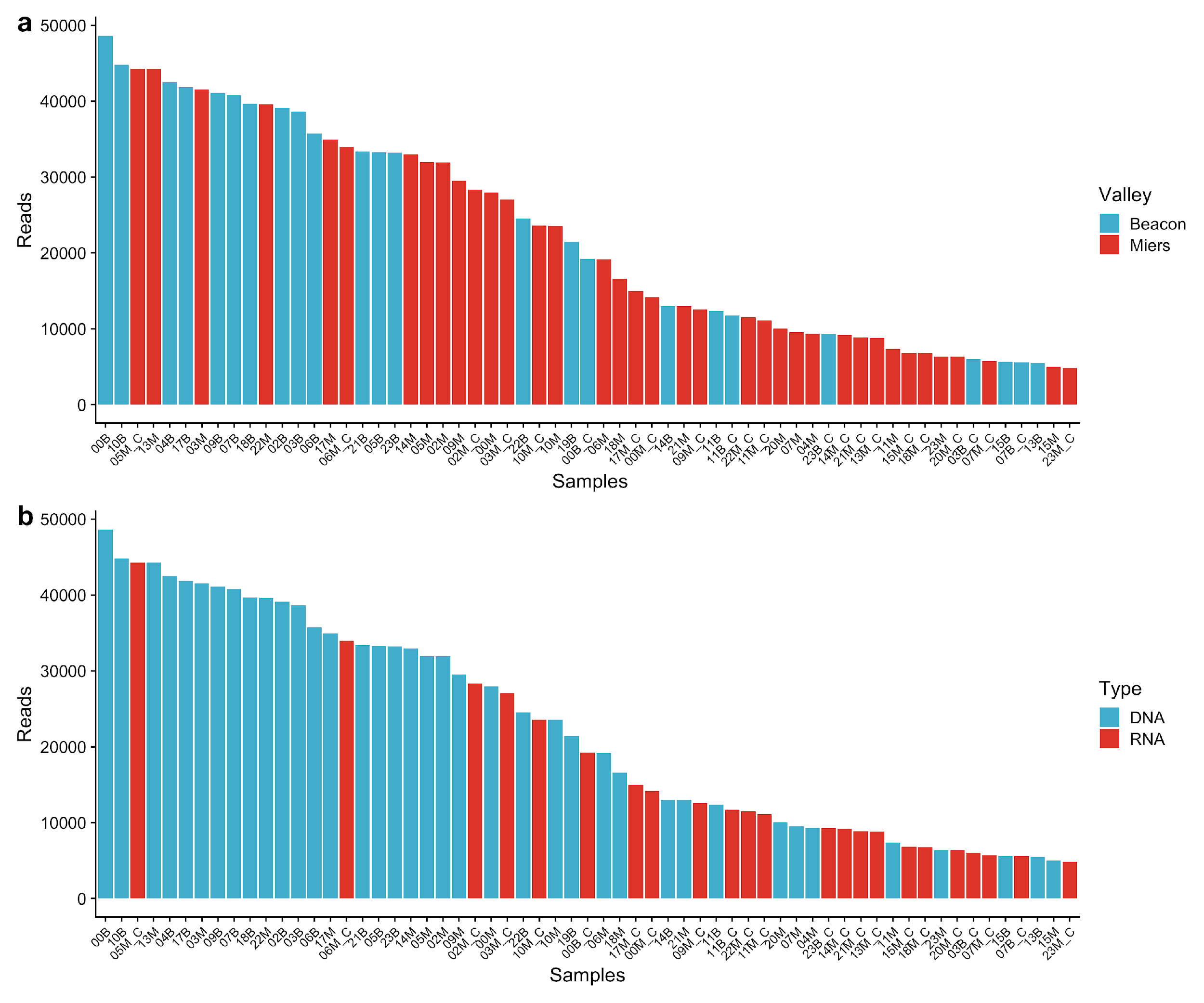


**Figure S1.** Number of reads in each sample coloured by **a** valley and **b** nucleic acid type. No obvious skewing could be seen in terms of number of sequences in both cases.


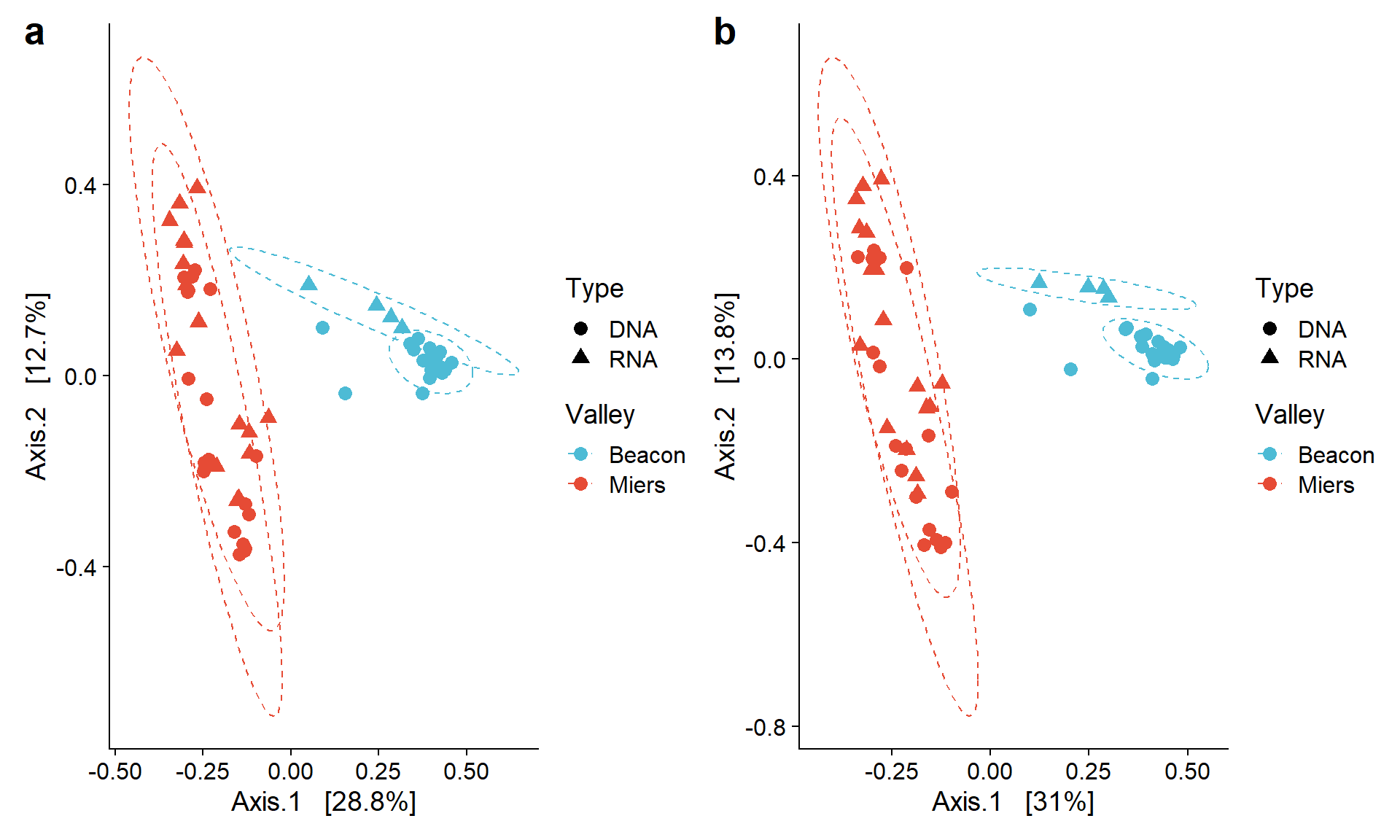


**Figure S2.** Comparison of **a** full and **b** filtered data sets. The filtered data set was obtained by removing ASVs with no more than 2 reads in at least 2 samples. The difference in variance explained by the PCoA first two axes in the full and filtered data sets was considered negligible (2 %). Ellipses are overlaid at the 0.95 level.


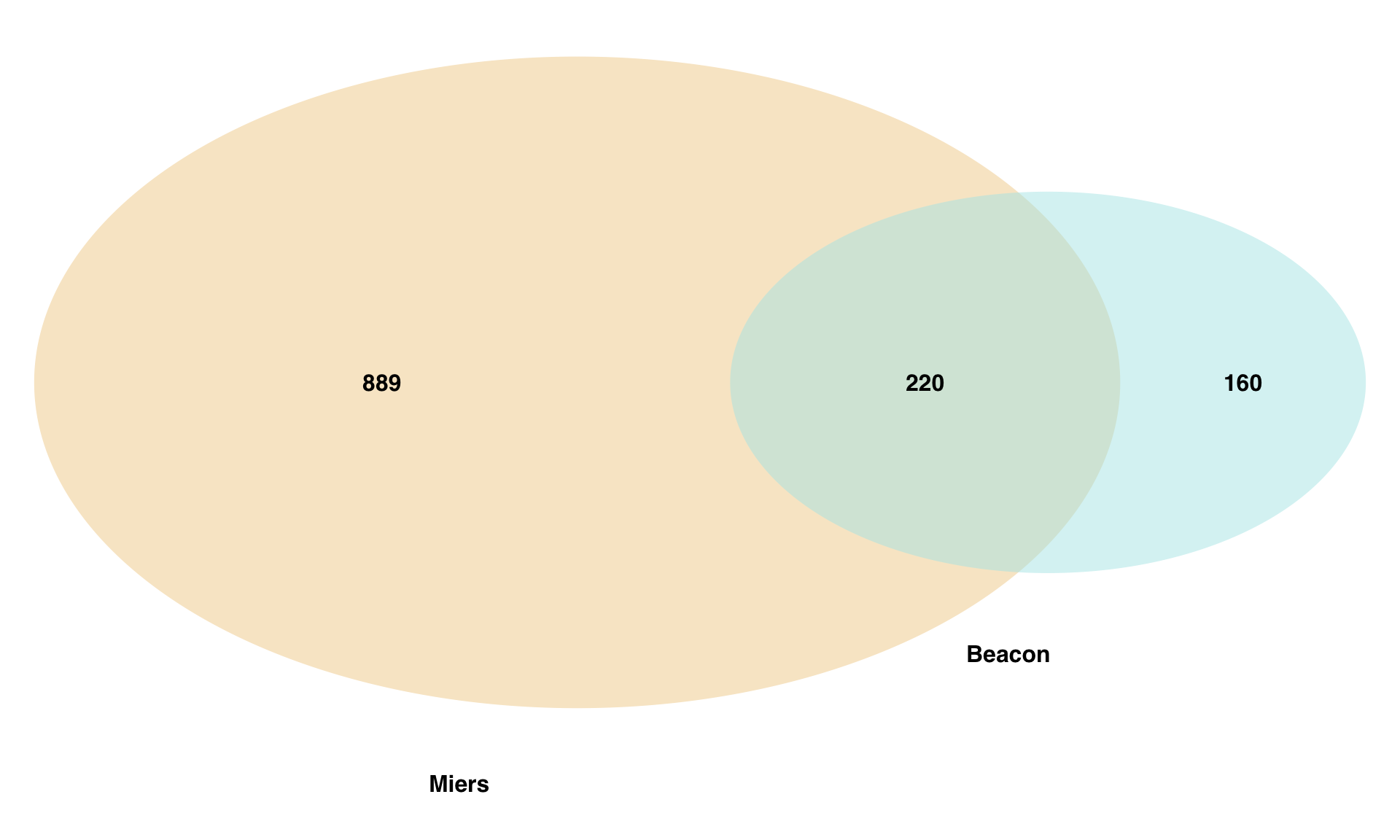


**Figure S3.** Number of ASVs shared between Beacon and Miers Valleys. Data are from both DNA and RNA-based samples.


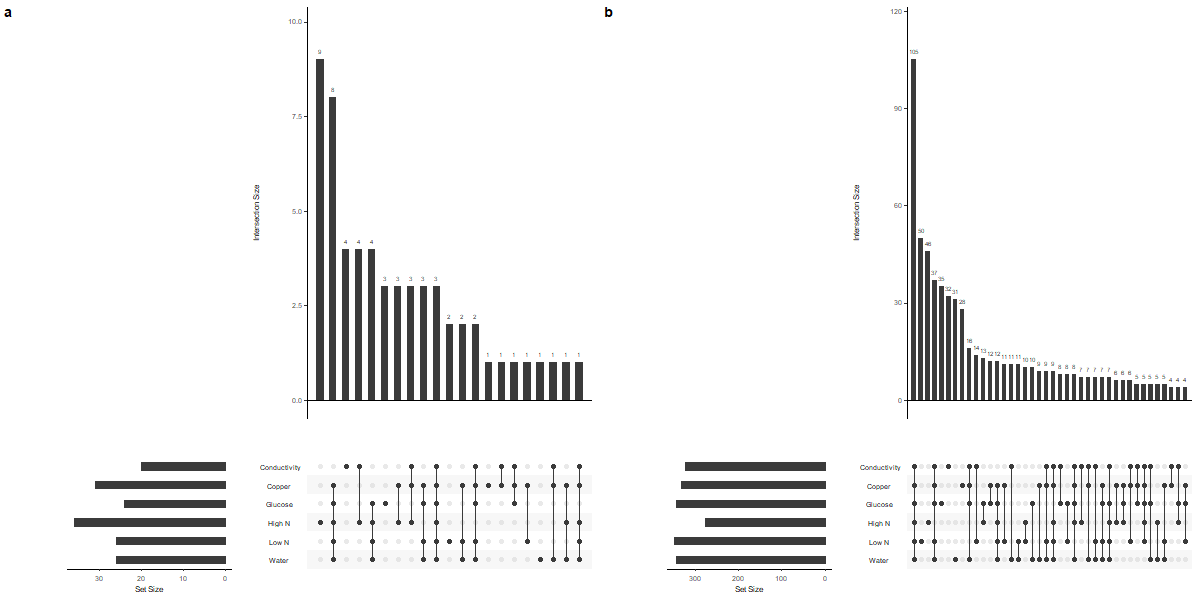


**Figure S4.** Number of rare ASVs shared by each disturbance in **a** Beacon Valley and **b** Miers Valley. Data are from DNA-based samples.

**Supplementary tables**

| **Table S1. Number of sequences from raw reads to data set for downstream analysis** | | | | | |
| --- | --- | --- | --- | --- | --- |
|  |  |  |  |  |  |
| Sample | Valley | Type | Raw | Filtered* | Final** |
| 00M | Miers | DNA | 63309 | 29425 | 27964 |
| 02M | Miers | DNA | 79088 | 33574 | 31898 |
| 03M | Miers | DNA | 113291 | 43457 | 41304 |
| 04M | Miers | DNA | 53561 | 10447 | 9190 |
| 05M | Miers | DNA | 84315 | 33372 | 31840 |
| 06M | Miers | DNA | 84563 | 35399 | 19125 |
| 07M | Miers | DNA | 56432 | 10740 | 9495 |
| 09M | Miers | DNA | 70394 | 30824 | 29459 |
| 10M | Miers | DNA | 56342 | 24692 | 23487 |
| 11M | Miers | DNA | 57111 | 8203 | 7301 |
| 13M | Miers | DNA | 85097 | 45963 | 44143 |
| 14M | Miers | DNA | 83258 | 34312 | 32808 |
| 15M | Miers | DNA | 35546 | 5741 | 4990 |
| 17M | Miers | DNA | 88151 | 36692 | 34874 |
| 18M | Miers | DNA | 82152 | 17680 | 16313 |
| 20M | Miers | DNA | 67011 | 11160 | 10001 |
| 21M | Miers | DNA | 69120 | 14051 | 13008 |
| 22M | Miers | DNA | 66466 | 24835 | 39586 |
| 23M | Miers | DNA | 45776 | 7423 | 6183 |
| 00M_C | Miers | RNA | 86296 | 42035 | 14169 |
| 02M_C | Miers | RNA | 63672 | 30348 | 28333 |
| 03M_C | Miers | RNA | 92333 | 28583 | 27042 |
| 05M_C | Miers | RNA | 122435 | 46759 | 44217 |
| 06M_C | Miers | RNA | 46827 | 12299 | 33756 |
| 07M_C | Miers | RNA | 32307 | 6609 | 5722 |
| 09M_C | Miers | RNA | 45649 | 13035 | 12565 |
| 10M_C | Miers | RNA | 46193 | 20443 | 23500 |
| 11M_C | Miers | RNA | 59129 | 12198 | 11103 |
| 13M_C | Miers | RNA | 55275 | 9764 | 8811 |
| 14M_C | Miers | RNA | 81030 | 10260 | 9191 |
| 15M_C | Miers | RNA | 44534 | 7597 | 6840 |
| 17M_C | Miers | RNA | 66326 | 16244 | 15005 |
| 18M_C | Miers | RNA | 49374 | 7333 | 6782 |
| 20M_C | Miers | RNA | 38762 | 6987 | 6354 |
| 21M_C | Miers | RNA | 43075 | 9635 | 8872 |
| 22M_C | Miers | RNA | 40325 | 15128 | 11474 |
| 23M_C | Miers | RNA | 36448 | 5474 | 4824 |
| 00B | Beacon | DNA | 87854 | 50421 | 48576 |
| 02B | Beacon | DNA | 76922 | 39944 | 39038 |
| 03B | Beacon | DNA | 81851 | 39743 | 37612 |
| 04B | Beacon | DNA | 101571 | 44050 | 41878 |
| 05B | Beacon | DNA | 61440 | 34287 | 33251 |
| 06B | Beacon | DNA | 73584 | 36284 | 34925 |
| 07B | Beacon | DNA | 88971 | 41356 | 40076 |
| 09B | Beacon | DNA | 83989 | 42712 | 41044 |
| 10B | Beacon | DNA | 81013 | 46307 | 44692 |
| 11B | Beacon | DNA | 104613 | 12707 | 12277 |
| 13B | Beacon | DNA | 50683 | 5701 | 5464 |
| 14B | Beacon | DNA | 81363 | 13620 | 13012 |
| 15B | Beacon | DNA | 40690 | 6554 | 5593 |
| 17B | Beacon | DNA | 87294 | 42653 | 41786 |
| 18B | Beacon | DNA | 79086 | 40376 | 38439 |
| 19B | Beacon | DNA | 65033 | 21971 | 21042 |
| 21B | Beacon | DNA | 67933 | 33986 | 33317 |
| 22B | Beacon | DNA | 50145 | 25105 | 24109 |
| 23B | Beacon | DNA | 87244 | 34425 | 32159 |
| 00B_C | Beacon | RNA | 100903 | 20292 | 19191 |
| 03B_C | Beacon | RNA | 27714 | 6392 | 6013 |
| 07B_C | Beacon | RNA | 27584 | 5846 | 5609 |
| 11B_C | Beacon | RNA | 46740 | 12239 | 11695 |
| 23B_C | Beacon | RNA | 44806 | 9845 | 9273 |
| * includes removal of chimeras | | |  |  |  |
| ** after removal of ASVs assigned to Eukaryota, Chloroplast, and Mitochondria | | | | | |
